# Comparison of African savanna elephant (*Loxodonta africana*) fatty acid profiles in whole blood, whole blood dried on blood spot cards, serum, and plasma

**DOI:** 10.7717/peerj.12650

**Published:** 2021-12-14

**Authors:** Jordan Wood, Larry J. Minter, Doug Bibus, Michael K. Stoskopf, Vivek Fellner, Kimberly Ange-van Heugten

**Affiliations:** 1 Animal Science, North Carolina State University, Raleigh, NC, United States of America; 2 Environmental Medicine Consortium and Department of Clinical Sciences, College of Veterinary Medicine, North Carolina State University, Raleigh, NC, United States of America; 3 North Carolina Zoo, Asheboro, NC, United States of America; 4 Lipid Technologies LLC, Austin, MN, United States of America

**Keywords:** African elephant, Fatty acids, Blood fractions, Dried blood spots

## Abstract

**Background:**

African elephants in managed care have presented differences in the balance between omega-3 and omega-6 fatty acids, a situation primarily thought to be due to dietary differences between the managed animals and their free-ranging counterparts. Because of this, circulating fatty acid status is included in routine monitoring of elephant health. A method of blood collection that requires only a few drops of whole blood, dried on filter paper (DBS) and can be used for analyzing full fatty acid profiles offers advantages in clinical application.

**Methods:**

This study compared the use of whole blood, and whole blood DBS, serum or plasma for use in evaluating circulating fatty acid composition in African savannah elephants. Samples from six African elephants (two males and four females) were collected during the same week at the NC Zoo, Asheboro, NC.

**Results:**

Results found only 2 of 36 individual fatty acids and none of the 10 fatty acid groupings were different when comparing the four blood fraction sample types to each other with Mann-Whitney U-Test pairwise comparisons. Myristic acid (14:0) was lower in the DBS samples than in whole blood, serum, and plasma and pentadecaenoic acid (15:1) was slightly more concentrated in DBS and whole blood.

**Discussion:**

Results indicate that fatty acid profile of serum, plasma, whole blood, and DBS are comparable in African elephants. The DBS method offers advantages in acquisition and handling and may be preferable to other methods in both routine health assessment of captive animals and field research on free ranging animals.

## Introduction

The optimal management and conservation of African savanna elephants (*Loxodonta africana*) in both free-ranging and managed environments requires better understanding of their metabolic and nutritional status. Free fatty acids, and the triglycerides they form, are critical for the health and function of a variety of body systems and are also biomarkers for many health concerns (Nagy & Tiuca, 2017). The importance of fatty acids includes cell membrane integrity and the production of energy for the body as well as impacts on inflammatory processes, cardiovascular, liver, pancreas, and retina health (Connor, 2000; Figueiredo et al., 2017; Nagy & Tiuca, 2017). Studies primarily in rodents have shown Omega-3 fatty acids lower inflammatory responses, reduce the risk of atherosclerosis, and improve pancreatic function by reducing insulin resistance and increase reproductive success (Connor, 2000; Figueiredo et al., 2017; Fritsche, 2006; Nagy & Tiuca, 2017). While understudied in other species, correlations between dietary omega-3 and omega-6 imbalances and atherosclerosis have been noted in several species, including African elephants (McCullaugh, 1972). Additionally, studies have found differences in circulating fatty acids when comparing free ranging *versus* managed animals for several species (Clauss et al., 2008; Clauss, Grum & Hatt, 2007; Dass et al., 2020; Dass et al., 2021; Schmidt et al., 2009).

Most data available on African elephant free fatty acids is only available from serum or plasma samples (Clauss et al., 2003; McCullagh, 1973; Moore & Sikes, 1967; Wood et al., 2020). Plasma and serum have been considered to better reflect the short-term circulating fatty acid status of animals while whole blood is thought to provide more information on the overall fatty acid status (Baylin et al., 2005; Hodson et al., 2013; Risé et al., 2007; Thomas Brenna et al., 2018). With differing opinions on the best blood fraction to collect for fatty acid analysis and concerns about sample collection, handling, and storage, researchers have begun to look to new blood collection methods for further answers.

Alternative methods of fatty acids analysis have been successfully used to analyze human blood with a few drops of dried whole blood on filter paper cards (Armstrong, Metherel & Stark, 2008; Bailey-Hall, Nelson & Ryan, 2008). These only require small volumes of whole blood to run full free fatty acid profiles and are more easily collected and stored in field research settings (Freeman et al., 2018). Because of this positive impact on field research, DBS cards have become more prevalent in wildlife research, but direct comparisons to serum, plasma, or liquid whole blood for a majority of species is lacking (Koutsos et al., 2021; Dass et al., 2020; Dass et al., 2021). This has led to the research question of how comparable DBS samples compare to more traditional samples such as liquid whole blood, serum, or plasma regarding fatty acid profiling.

The goal of this research was to: compare the results of fatty acid analysis of DBS samples to (a) traditionally collected whole blood, (b) serum, and (c) plasma of a cohort of managed elephants maintained on a known diet.

## Materials & Methods

### Animals and diets

Six adult African elephants (two males and four females) managed at the North Carolina (NC) Zoo, Asheboro, NC, USA were sampled between 8:30 and 9:30 AM within one week during July 2020. This study was approved by the NC Zoo Animal Research Committee. These animals were fed a diet of Mazuri^®^ Hay Enhancer™, fresh cut browse, timothy hay, and daytime pasture access for grazing.

### Sample collection and analyses

Blood samples were taken under veterinary supervision *via* auricular veins during routine monthly wellness exams. Blood was placed into (1) untreated red top vacutainer tubes, (2) lithium heparin green top vacutainer tubes, (3) serum separator red and black top vacutainer tubes, and (4) plasma separator tubes with lithium heparin light green top vacutainer tubes (Becton, Dickinson and Company, Franklin Lakes, NJ, USA). All elephants were trained with positive reinforcement for regular blood collections, so restraints were not used. Previous serum samples collected and analyzed for fatty acids in 2016–2017 by Wood et al. (2020) were not included in the current study and serum samples collected in this study are new and are not part of the work published in 2020. Whole blood collected in the untreated vacutainers were transferred to Perkin-Elmer Spot Saver cards (Perkin-Elmer, Waltham, MA, USA) using four spots of approximately 80 µL of whole blood (approximately 320 µL per card). Samples collected in serum and plasma separator tubes were centrifuged and transferred to cryovials. Cryovials of whole blood, plasma, and serum as well as dried blood spot (DBS) cards were frozen at −80 °C within 3 h of collection and stored for approximately 2 months before being shipped on dry ice to Lipid Technologies (Austin, MN, USA) for a simultaneous full fatty acid profile analysis including 36 individual fatty acids and 10 fatty acid groups (Table 1). Using established methods, samples were transmethylated with acidified methanol and the fatty acid methyl esters were quantified by area percent as analyzed on routine gas chromatography (https://lipidlab.com/services/; Koutsos et al., 2021). Values are provided on a percent of total fatty acids present.

**Table 1:** Fatty acid (% of total fatty acids) profile averages and standard deviations (sd) of dry blood spot cards (DBS) (*n* = 6), whole blood (*n* = 6), plasma (*n* = 6), and serum (*n* = 6) samples from managed african savanna elephants (*Loxodonta africana*)^1–6^.

**Sample type**	**DBS**	**Whole blood**	**Plasma**	**Serum**
**Individual fatty acid**	**Average (SD)**	**Average (SD)**	**Average (SD)**	**Average (SD)**
Lauric acid (12:0)	ND	ND	ND	ND
Myristic acid (14:0)^7^	0.74^a,x,¶^ (0.31)	1.9^y^ (0.28)	2.0^b^ (0.46)	1.7^‡^ (0.34)
Myristoleic acid (14:1)	0.00 (0.00)	0.07 (0.04)	0.02 (0.05)	0.03 (0.05)
Pentadecylic acid (15:0)	0.00 (0.00)	0.79 (0.12)	0.52 (0.15)	1.01 (0.49)
Pentadecanoic acid (15:1)	0.61^a,¶^ (0.10)	0.57^∘^ (0.19)	0.26^b,^^§^ (0.08)	0.34^‡^ (0.19)
Palmitic acid (16:0)	17.8 (1.98)	25.4 (2.83)	19.2 (3.04)	18.9 (1.67)
9-hexadecaenoic acid (16:1w5)	ND	ND	ND	ND
Palmitoleic acid (16:1w7)	3.5 (0.85)	3.8 (0.92)	2.9 (1.22)	3.1 (1.12)
Margaric acid (17:0)	ND	ND	ND	ND
Heptadecaenoic acid (17:1)	ND	ND	ND	ND
Stearic acid (18:0)	10.1 (0.94)	11.8 (1.92)	9.5 (0.84)	8.9 (1.31)
13-octadecaenoic acid (18:1w5)	ND	ND	ND	ND
Vaccenic acid (18:1w7)	ND	ND	ND	ND
Oleic acid (18:1w9)	23.2 (2.46)	28.4 (2.08)	18.6 (3.04)	19.4 (3.76)
Linoleic acid (18:2w6)	18.6 (2.27)	13.2 (2.56)	22.5 (3.80)	22.6 (4.36)
γ-linolenic acid (18:3w6)	0.80 (0.38)	0.23 (0.10)	0.89 (0.40)	0.80 (0.40)
α-linolenic acid (18:3w3)	3.4 (0.47)	1.8 (0.47)	3.9 (0.78)	3.5 (0.68)
Stearidonic acid (18:4w3)	0.32 (0.08)	0.10 (0.04)	0.25 (0.13)	0.22 (0.09)
Arachdic acid (20:0)	0.34 (0.14)	0.32 (0.08)	0.14 (0.02)	0.11 (0.05)
Eicosenoic acid (20:1w9)	0.00 (0.00)	0.04 (0.04)	0.00 (0.00)	0.00 (0.00)
Paullinic acid (20:1w7)	0.99 (0.14)	0.63 (0.26)	0.53 (0.33)	0.36 (0.33)
Eicosenoic acid (20:2w6)	0.29 (0.11)	0.25 (0.10)	0.35 (0.11)	0.36 (0.11)
Mead acid (20:3w9)	0.04 (0.05)	0.01 (0.02)	0.05 (0.03)	0.00 (0.00)
h-γ-linolenic acid (20:3w6)	3.6 (0.48)	2.2 (0.50)	3.6 (0.83)	3.7 (0.75)
Arachidonic acid (20:4w6)	9.0 (1.31)	4.1 (1.06)	8.7 (2.38)	8.8 (2.21)
Eicosatrienoic acid (20:3w3)	0.11 (0.04)	0.10 (0.02)	0.15 (0.04)	0.17 (0.07)
Eicosatetraenoic acid (20:4w3)	0.57 (0.35)	0.39 (0.17)	0.93 (0.37)	0.97 (0.34)
Eicosapentaenoic acid (20:5w3)	2.1 (0.66)	0.8 (0.31)	1.9 (0.87)	1.7 (0.65)
Behenic acid (22:0)	0.67 (0.18)	0.04 (0.03)	0.03 (0.04)	0.01 (0.02)
Erucic acid (22:1w9)	0.11 (0.17)	0.71 (0.17)	0.65 (0.08)	0.74 (0.37)
Docosatetraenoic (adrenic) acid (22:4w6)	0.35 (0.10)	0.38 (0.20)	0.35 (0.07)	0.38 (0.06)
DPA (osbond acid) (22:5w6)	0.09 (0.12)	0.33 (0.15)	0.21 (0.14)	0.15 (0.05)
DPA (clupanodonic acid) (22:5w3)	1.5 (0.39)	0.7 (0.31)	1.6 (0.55)	1.6 (0.50)
Lignoceric acid (24:0)	0.30 (0.08)	0.09 (0.08)	0.04 (0.04)	0.03 (0.02)
Docosahexaenoic acid (22:6w3)	0.36 (0.13)	0.72 (0.37)	0.24 (0.10)	0.22 (0.08)
Nervonic acid (24:1)	0.07 (0.07)	0.08 (0.08)	0.02 (0.04)	0.11 (0.05)
**Fatty acid groups**	**Average (SD)**	**Average (SD)**	**Average (SD)**	**Average (SD)**
Saturates	30.5 (1.83)	40.4 (1.45)	31.4 (3.02)	30.7 (2.14)
Monoenes	25.0 (2.42)	30.5 (2.13)	20.1 (3.29)	21.0 (4.17)
Poly unsaturated fatty acids (PUFA)	41.0 (3.20)	25.3 (3.13)	45.6 (6.70)	45.2 (6.73)
Highly unsaturated fatty acids (HUFA)	17.6 (2.74)	9.7 (2.21)	17.8 (4.72)	17.7 (3.99)
Total w3 fatty acids	8.3 (1.00)	4.5 (0.81)	9.0 (1.51)	8.4 (0.91)
Total w6 fatty acid	32.7 (2.83)	20.7 (2.84)	36.6 (5.98)	36.8 (6.20)
Total w9 fatty acids	23.4 (2.41)	29.3 (2.27)	19.3 (3.08)	20.3 (3.98)
w6/w3 fatty acid ratio	4.0 (0.51)	4.7 (0.90)	4.1 (0.74)	4.4 (0.64)
Omega 3 HUFA	25.6 (3.73)	26.8 (4.27)	26.8 (4.67)	25.9 (3.76)
Omega 6 HUFA	74.4 (3.73)	73.2 (4.27)	73.2 (4.67)	74.2 (3.76)

**Notes.**

1 Fatty acids that were not in high enough concentration to be quantified included: lauric acid (12:0), 9-hexadecenoic acid (16:1w5), margaric acid (17:0), heptadecenoic acid (17:1), vaccenic acid (18:1w7), and 13-octadecenoic acid (18:1w5).

2 Differing superscripts (^a,b^) in averages columns are significantly different at (α = 0.05, U-statistic = 5) for DBS compared to plasma.

3 Differing superscripts (^x,y^) in averages columns are significantly different at (α = 0.05, U-statistic = 5) for DBS compared to whole blood.

4 Differing superscripts (^¶,‡^) in averages columns are significantly different at (α = 0.05, U-statistic = 5) for DBS compared to serum.

5 Differing superscripts (^¶, ∘^) in averages columns are significantly different at (α = 0.05, U-statistic = 5) for plasma compared to whole blood.

6 Differing superscripts (^¥,£^) in averages columns are significantly different at (α = 0.05, U-statistic = 5) for serum compared to whole blood.

7 Outlier from DBS samples was removed for mean and SD calculations thus DBS *n* = 5 (U-statistic = 3).

### Statistics

Statistical analysis was conducted to compare DBS, whole blood, serum, and plasma to each other using pairwise comparisons by the Mann–Whitney U-test with an α = 0.05 and a *U* test statistic of 5. Pairwise comparisons included (1) DBS compared to whole blood (*U*-statistic = 5), (2) DBS compared to serum (*U*-statistic = 5), (3) DBS compared to plasma (*U*-statistic = 5), (4) whole blood compared to serum (*U*-statistic = 5), (5) whole blood compared to plasma (*U*-statistic = 5), and (6) serum compared to plasma (*U*-statistic = 5). Because the sample size was small (*n* = 6) and the use of nonparametric statistics, *p*-values are not provided in this article because this method of statistical analysis leads to *p*-values that are inaccurate and do not provide valuable information. Instead of *p*-values, the Mann–Whitney *U*-test uses a critical value *U*-statistic for comparison, when the calculated value is less than or equal to the critical value *U*-statistic then we fail to reject the null hypothesis and there are no differences.

## Results

Of the 36 individual fatty acids and 10 fatty acids groups identified, 30 individual and 10 fatty acid groups were quantifiable (Table 1). The six fatty acids that were not present in sufficient quantity to be reliably quantified within any of the blood sample types were lauric acid (12:0), 9-hexadecenoic acid (16:1w5), margaric acid (17:0), heptadecenoic acid (17:1), vaccenic acid (18:1w7), and 13-octadecenoic acid (18:1w5).

Pairwise comparisons of DBS and whole blood, DBS and serum, DBS and plasma, whole blood and serum, whole blood and plasma, and serum and plasma using the Mann–Whitney U-Test, found differences between sample type only for myristic acid (14:0) and pentadecaenoic acid (15:1). Data for myristic acid (14:0) initially showed a lower concentration present in the DBS samples than in whole blood, serum, and plasma with a very large variability. This was apparently due to one sample which was an obvious outlier. When statistics were run excluding this outlier DBS card data for myristic acid was tighter but much lower than identified with any on the other types of blood samples. The differences identified for pentadecaenoic acid (15:1) concentrations were complicated by variability among the whole blood results and serum results. These variations were much greater than seen for plasma and particularly for DBS sample results (Table 1). No notable differences between data from frozen whole blood samples were seen when comparing the animals by age (approximately ages 14 to 48 years) or sex.

## Discussion

Results of this study found DBS to be an acceptable method when compared to the analysis of other blood fractions examined for both individual fatty acids and fatty acid groups with the possible exception of the fatty acids, myristic and pentadecaenoic acid. This finding is consistent with studies on human blood that have found that DBS is a useful and comparable collection method for fatty acids (Armstrong, Metherel & Stark, 2008; Bailey-Hall, Nelson & Ryan, 2008; Thomas Brenna et al., 2018). Visual differences of interest included the lack of myristoleic acid (14:1) and pentadecylic acid (15:0) found in the DBS samples. Myristoleic acid concentrations were very low across all sample types and only a few were detectable above baseline. It is possible that differences seen between sample types was due to residue from the filter paper or external contamination, but more likely the low concentrations present were below reliable detection limits considering the expected error in sample extraction methods. The statistically significant differences for myristic acid and pentadecanoic acid (15:1) are hypothesized have been related to challenges with elution from the DBS card.

Values found in this study were similar to previous samples collected and analyzed by the authors with the same six African elephants (Wood et al., 2020). These values were also comparable to serum and plasma values collected in free-ranging elephants (Clauss et al., 2003; McCullagh, 1973; Moore & Sikes, 1967).

Results from this study were favorable for cross comparisons of important individual fatty acids and fatty acid groups including traditional essential fatty acids: α-linolenic acid, linoleic acid, critical fatty acids: eicosapentaenoic acid (EPA) and docosahexaenoic acid (DHA), and important fatty acid groups: omega-3 fatty acids, polyunsaturated fatty acids (PUFAs), and the omega-6: omega-3 ratio. As mentioned in the introduction, in managed settings, it has been noted that herbivores often have higher levels of omega-6 fatty acids in their diet and circulation leading to an inappropriate omega-6: omega-3 ratio when compared to free-ranging animals (Clauss et al., 2003; Clauss, Grum & Hatt, 2007). This could be relevant to problems with obesity in elephants in managed care (Morfeld et al., 2016). Being able to track circulating omega-3 fatty acids, especially EPA and DHA in elephants and potentially other megavertebrates using the simpler DBS collection of a few drops of blood would greatly facilitate better monitoring across institutions.

## Conclusions

Data provided in this study supports the hypothesis that fatty acid composition of whole blood, plasma, and serum are very similar in African savanna elephants. Fatty acid results from DBS samples provide a reasonably comparable approach to liquid whole blood samples, which are more difficult to store and ship.

## Supplemental Information

10.7717/peerj.12650/supp-1 Supplemental Information 1 Raw Data for Statistical Analysis of Differences between Whole Blood, Whole Blood Dried Blood Spots, Serum, and Plasma Collected from Six African Savanna Elephants

## Additional Information and Declarations

### Data Availability

The following information was supplied regarding data availability: The raw data is available in the Supplemental File.
